# Cerebrospinal fluid proteomic profile of frailty: Results from the PROLIPHYC cohort

**DOI:** 10.1111/acel.14168

**Published:** 2024-05-02

**Authors:** Sophie Guillotin, Amit Fulzele, Alexandra Vallet, Anne Gonzalez de Peredo, Emmanuelle Mouton‐Barbosa, Philippe Cestac, Sandrine Andrieu, Odile Burlet‐Schiltz, Nicolas Delcourt, Eric Schmidt

**Affiliations:** ^1^ Aging‐MAINTAIN Research Team, Center for Epidemiology and Research in POPulation Health (CERPOP) University of Toulouse Toulouse France; ^2^ Poison Control Center Toulouse University Hospital Toulouse France; ^3^ Institute of Pharmacology and Structural Biology (IPBS) University of Toulouse, CNRS, University of Toulouse III (Paul Sabatier (UT3) Toulouse France; ^4^ Biological Tissue and Surface Engineering Department INSERM U1059 Sainbiose, Ecole Des Mines of Saint‐Etienne Saint‐Etienne France; ^5^ Department of Clinical Pharmacy Toulouse University Hospital Toulouse France; ^6^ Department of Epidemiology and Public Health Toulouse University Hospital Toulouse France; ^7^ IHU HealthAge Toulouse France; ^8^ Toulouse NeuroImaging Center (ToNIC) University of Toulouse, INSERM UPS Toulouse France; ^9^ Department of Neurosurgery Toulouse University Hospital Toulouse France; ^10^ Present address: Institute of Molecular Biology University of Mainz Mainz Germany

**Keywords:** central nervous system, cerebrospinal fluid, frailty, frailty prediction, proteomics

## Abstract

Frailty is a clinical state reflecting a decrease in physiological reserve capacities, known to affect numerous biological pathways and is associated with health issues, including neurodegenerative diseases. However, how global protein expression is affected in the central nervous system in frail subject remains underexplored. In this post hoc cross‐sectional biomarker analysis, we included 90 adults (52–85 years) suspected of normal pressure hydrocephalus (NPH) and presenting with markers of neurodegenerative diseases. We investigated the human proteomic profile of cerebrospinal fluid associated with frailty defined by an established cumulated frailty index (FI, average = 0.32), not enriched for neurology clinical features. Using a label‐free quantitative proteomic approach, we identified and quantified 999 proteins of which 13 were positively associated with frailty. Pathway analysis with the top positively frailty‐associated proteins revealed enrichment for proteins related to inflammation and immune response. Among the 60 proteins negatively associated with frailty, functional pathways enriched included neurogenesis, synaptogenesis and neuronal guidance. We constructed a frailty prediction model using ridge regression with 932 standardized proteins. Our results showed that the “proteomic model” could become an equivalent predictor of FI in order to study chronological age. This study represents the first comprehensive exploration of the proteomic profile of frailty within cerebrospinal fluid. It sheds light on the physiopathology of frailty, particularly highlighting processes of neuroinflammation and inhibition of neurogenesis. Our findings unveil a range of biological mechanisms that are dysregulated in frailty, in NPH subjects at risk of neurodegenerative impairment, offering new perspectives on frailty phenotyping and prediction.

## Graphical abstract

To gain insight into the central nervous system with a view to better understanding the pathophysiology of frailty, we undertook a statistical analysis linking the frailty index to proteomic data derived from quantitative analysis of cerebrospinal fluid from older patients. First, the results reveal an association between frailty and the processes of neuroinflammation, immunosenescence and neurodegeneration in the brain. Second, we showed that the “proteomic model” could become an equivalent predictor of FI in order to study chronological age.

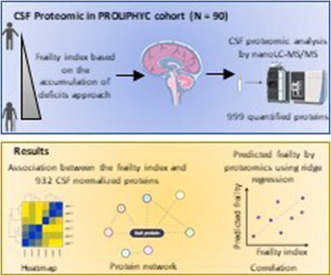

**Abbreviations**
AD: alzheimer disease
CNS: central nervous system
CSF: cerebrospinal fluid
FI: frailty index
IPSS: international prostate symptome score
MMSE: mini mental state examination
NPH: normal pressure hydrocephalus
PD: parkinson disease
R0: CSF outflow resistance
UPDRS: unified parkinson's disease rating scale

## 1 INTRODUCTION

Frailty is defined as a clinical syndrome. It reflects a decrease in physiological reserve capacities that alters the mechanisms of adaptation to stress. Its clinical phenotype is modulated by biological, physiological, social, and environmental factors. It is currently measured as the accumulation of declines in multiple systems which are trusted to be leading to the increased vulnerability to stress (Fried et al., 2001; Nourhashémi et al., 2001). Estimating frailty level is of interest as it has been shown to be associated with increased risk of disability, falls, hospitalization, and death.

Concerning the central nervous system (CNS) pathologies, frailty has been associated with cognitive decline, dementia (Avila‐Funes et al., 2012; Buchman et al., 2014; Solfrizzi et al., 2013), neuropathological features such as beta‐amyloid and tau accumulation (Wallace et al., 2018), and brain atrophy (Kant et al., 2018).

Our latest research showed an association between frailty and abnormal mechanical properties of the CNS, thereby implying a major role of the CNS in this context (Vallet et al., 2020). Cognitive impairment has emerged as a major contributing factor to susceptibility to adverse outcomes, as supported by Cesari et al. (2013). This relationship may find illustration through various structural parameters, which is associated with frailty, such as alterations in brain volume and microstructural integrity (Taylor et al., 2023). However, specific CNS biomarkers for frailty remain understudied, with only one study indicating a correlation between lower BDNF concentrations and elevated frailty rates in plasma samples from non‐frail and pre‐frail elderly women (Coelho et al., 2012). Paired with the biomechanical parameters, this underscores the hypothesis regarding the potential significance of neurological signaling pathways in the pathophysiology of frailty (Cardoso et al., 2018).

Cerebrospinal fluid (CSF) is the extracellular fluid of the CNS, derived from the choroid plexus, and from interstitial fluid in gray and white matter. CSF is an ideal medium since its composition reflects the molecular environment of both neuronal and glial cells within the CNS. Any changes in CSF composition can provide valuable insights into brain pathophysiology, including inflammation, infection, or neurodegeneration. Given these findings, it is compelling to probe the biological signatures that characterize CNS impairments associated with frailty.

To achieve this objective, proteomic analysis of CSF emerges as a promising avenue. Proteomic analysis now allows multiplexed analyses rather than an individual biomarker to characterize a biological profile of this complex and dynamic frailty syndrome (Picca et al., 2022). It is also a technique of choice for studying the complete protein content of a biological sample. This technique has already proven its relevance in the discovery of protein signatures of frailty. While several studies have explored the plasma proteomic profile of frailty (Landino et al., 2021; Lin et al., 2017; Liu et al., 2023; Mitchell et al., 2023; Perry et al., 2023; Sathyan et al., 2020; Shamsi et al., 2012; Verghese et al., 2021), highlighting pathways associated with frailty such as the inflammatory response, coagulation, and lipid metabolism, there is a noticeable gap in the literature regarding the CSF proteomic profile of frailty. Therefore, CSF proteomics positions itself as a preferred technique for gaining a comprehensive understanding of the cerebral pathogenic pathways that are intertwined with frailty.

In this posthoc cross‐sectional biomarker analysis, we included 90 adults (52–85 years) suspected of normal pressure hydrocephalus (NPH) and presenting with markers of Parkinson disease (PD), Alzheimer disease (AD), and vascular dementia. Lumbar puncture is required for tap test and important for diagnosing NPH, providing a significant opportunity to analyze the CSF proteome in frail patients. Our aim was to elucidate the protein composition of CSF in association with frailty. We hypothesized that in this sample, (a) frailty would be associated with a CSF protein signature and (b) frailty predicted by CSF proteomics would serve as a reliable predictor of chronological age. To address these hypotheses, we conducted the first shotgun quantitative CSF proteomic analysis. Together, our results enabled us to highlight a window within CNS providing biological insights into the statistical correlations between frailty and neurological impairments. These contributions can open up new avenues for investigating and understanding frailty in a neurological context.

## 2 MATERIALS AND METHODS

### 2.1 PROLIPHYC cohort

One hundred subjects suspected of NPH (46 women and 54 men aged 52–92 years) were prospectively included in the PROLIPHYC cohort between 2013 and 2015 at Toulouse University Hospital. The suspicion of NPH was based on established guidelines that is, the presence of (1) clinical symptoms of gait or balance disturbance, cognitive impairment or urinary disorders, (2) neuroimaging evidence of ventriculomegaly (i.e., Evans Index >0.3), and (3) narrow callosal angle, temporal horn enlargement or periventricular signal changes (Relkin et al., 2005). The study was approved by the competent authority and registered at ClinicalTrials.gov under no NCT02016352. The local ethics committee approved the study. All subjects gave their written informed consent.

### 2.2 Frailty assessment

Frailty was quantified by means of a frailty index (FI) based on the deficit accumulation approach, which has been widely validated across many settings and populations (Rockwood & Mitnitski, 2011; Searle et al., 2008). The FI was defined as the ratio, ranging from 0 (not frail) to 1 (very frail), between the number of health deficits presented by the individual and the total number of deficits considered. A total of 40 variables were selected to measure the deficits (Data S3—Supplemental Material, Table S1) and validated in two previous studies (Guillotin et al., 2022; Vallet et al., 2020).

### 2.3 Clinical characterization of the population

NPH is an important differential diagnosis of neurodegenerative diseases (Skalický et al., 2020). NPH refers to a complex brain disorder characterized by ventricular enlargement and the classic Hakim's triad of gait and balance difficulties, urinary incontinence, and cognitive impairment. Modern approach of NPH is moving away from focusing in an isolated neurological problem (Manet et al., 2023). In order to better characterize NPH heterogenous population, we gauged in every patient amyloid and vascular load, dopaminergic deficiency and resistance to CSF outflow (Data S3—Supplemental Material).

Patients underwent a structured clinical evaluation that included the following rating scales: second part of Unified Parkinson's Disease Rating Scale (UPDRS), International Prostate Symptom Score (IPSS), and Mini Mental State Examination (MMSE). We utilized moderate severity cutoff values to assess our patients. Specifically, we defined patients as having disabilities if their score on the second part of the UPDRS exceeded 16 (Rodriguez‐Blazquez et al., 2013), their IPSS score exceeded 7 (Barry et al., 1992), and their MMSE score was lower than 21 (National Institute for Health and Care Excellence, 2018).

### 2.4 Proteomic assessment

For every patient lumbar puncture was performed to withdraw CSF sample and infusion test. CSF sample was transported to the biochemistry laboratory in polypropylene tube on ice to be centrifuged, aliquoted and stored at −80°C. All lumbar punctures have been performed by a single investigator (ES) and a standardized procedure was applied to minimize variability.

Then CSF samples (1.4 mL) were unfrozen and concentrated on centrifugal units (Vivacon 500, 10 kDa cutoff, Sartorius) at 4°C. For each sample, depletion of 12 high abundant proteins (α1‐Acid Glycoprotein, α1‐Antitrypsin, α2‐Macroglobulin, Albumin, Apolipoprotein A‐I, Apolipoprotein A‐II, Fibrinogen, Haptoglobin, IgA, IgG, IgM, and Transferrin) was performed using single‐use spin columns containing immobilized antibodies (Pierce™ Top 12 Abundant Protein Depletion Spin Columns, Thermo Fisher Scientific), in order to reduce the dynamic range of protein concentration and improve the identification and quantification of low‐abundant proteins by mass spectrometry. Protein samples were lyophilized and resuspended in 6 M urea, 0.1 M Tris/HCl buffer pH 8. After protein assay, an equal amount of protein for each sample (20 μg) was reduced with 10 mM DTT and alkylated with 20 mM iodoacetamide. Protein were then first digested with 1% LysC for 5 h at a final concentration of 4 M urea, and then with 1% trypsin, overnight, after dilution of urea at 1 M. Resulting peptides were desalted on Sep‐Pak tC18 cartridges (Waters), vacuum dried, and reconstituted in 22 μL of 2% acetonitrile, 0.05% trifluoroacetic acid (TFA). Peptides were analysed by nanoscale liquid chromatography coupled to tandem mass spectrometry (nanoLC‐MS/MS) using an UltiMate 3000 RSLCnano system coupled to an Orbitrap Q‐Exactive Plus mass spectrometer (Thermo Fisher Scientific). Peptides (5 μL) were trapped and desalted on a C18 precolumn (300 μm inner diameter × 5 mm, Thermo Fisher Scientific) at a flow rate of 20 μL/min, then separated on an analytical C18 column (75 μm inner diameter × 50 cm, in‐house packed with 3 μm Reprosil C18) equilibrated in 95% solvent A (5% acetonitrile and 0.2% formic acid) and 5% solvent B (80% acetonitrile and 0.2% formic acid). Peptides were eluted using a 5%–50% gradient of solvent B over 200 min at a flow rate of 350 nL/min. The mass spectrometer was operated in data‐dependent acquisition mode with the Xcalibur software. MS survey scans were acquired on the 350–2000 *m/z* range with a resolution of 70,000 and an AGC target of 3e6. The 10 most intense ions were selected for fragmentation by high energy collision induced dissociation, and the resulting fragments were analysed at a resolution of 17,500, using an AGC target of 1e5 and a maximum fill time of 50 ms. Dynamic exclusion was used within 30 s to prevent repetitive selection of the same peptide. At least 2 replicate nanoLC‐MS/MS analytical runs were performed for each sample.

Raw MS files were processed with MaxQuant software (version 1.5.) for database search with the Andromeda search engine and quantitative analysis. Data were searched against human entries of the UniProt KB protein database (release UniProtKB/Swiss‐Prot version of January 2016, 20,189 entries), and the set of common contaminants provided by MaxQuant. Carbamidomethylation of cysteines was set as a fixed modification, whereas oxidation of methionine and protein N‐terminal acetylation were set as variable modifications. Specificity of trypsin digestion was set for cleavage after K or R, and two missed trypsin cleavage sites were allowed. The precursor mass tolerance was set to 20 ppm for the first search and 4.5 ppm for the main Andromeda database search. Minimum peptide length was set to seven amino acids, and minimum number of unique or razor peptides was set to one for validation. Andromeda results were validated by the target decoy approach using a reverse database, with a false discovery rate set at 1% at both PSM (peptide sequence match) and protein level. For label‐free relative quantification of the samples, the match between runs option of MaxQuant was enabled with a match time window of 1 min, to allow cross‐assignment of MS features detected in the different runs, after alignment of the runs with a time window of 20 min. Protein quantification was based on unique and razor peptides. The minimum ratio count was set to 1 for LFQ calculation. LFQ values from replicate MS injections of the same subject were averaged, and missing values were imputed with a low‐intensity value reflecting the noise background, randomly drawn from a normal distribution using the Perseus software version 1.5.

### 2.5 Statistical analysis

Descriptive analysis of quantitative parameters was performed with mean ± standard deviation (SD) and qualitative parameters with percentage.

The means of the observed intensity values were normalized using the square root mathematical adjustment. Outliers were removed using a density plot (average of removed outliers: 1.1%). To ascertain normality, protein abundance was assessed by histograms and Lilliefors test. In total, 932 normalized proteins were used in the statistical analysis.

The primary objective of this study was to identify the association between proteins and FI using Pearson regression analysis. Analyses were adjusted for age and sex. The linear model is FI ~ β0 + β1 protein level + β2 age + β3 sex + ε. Multiple test correction was performed, and Benjamini–Hochberg‐corrected *p* < 0.05 were considered statistically significant. To illustrate this, a heatmap was constructed. For better understanding, protein abundances and age were mean‐centered and standardized within the heatmap.

The second objective was to construct a penalized regression model using the R package glmnet (Friedman et al., 2010). First, the cohort was divided into two groups, 45 subjects for the training set, and 45 subjects for the validation set. Participants in the training set were selected using stratified random sampling method: selection within each stratum of 0.05 FI. Second, FI was regressed on 932 log‐transformed protein abundances. Third, using the cv‐glmnet function, the lambda value was selected on the basis of a 10‐fold cross‐validation using the training set. The alpha value of 0 was chosen for the ridge regression.

Statistical analyses were performed using R version 4.0.2.

### 2.6 Pathway analysis

Pathway analysis was performed using frailty‐associated proteins to uncover the biological pathways related to FI. This analysis was carried out using STRING (www.string‐db.org) with the significant proteins from the Pearson regression (Szklarczyk et al., 2019). The STRING network analysis resulted in a list of biological processes. The enrichment was measured by counts in network (“proteins in our network out of the total protein in this network”), the strength which describes how large the enrichment effect is log10 (observed proteins/expected proteins) and the False Discovery Rate (FDR) which describes how significant the enrichment is (*p*‐value adjusted by Benjamini–Hochberg procedure). Other networks were checked to validate previous pathway analysis using Reactome (www.reactome.org) (Gillespie et al., 2022) and Gene Ontology (www.geneontology.org) (Ashburner et al., 2000; Gene Ontology Consortium, 2021).

## 3 RESULTS

### 3.1 Clinical characteristic of the study population

**FIGURE 1:**
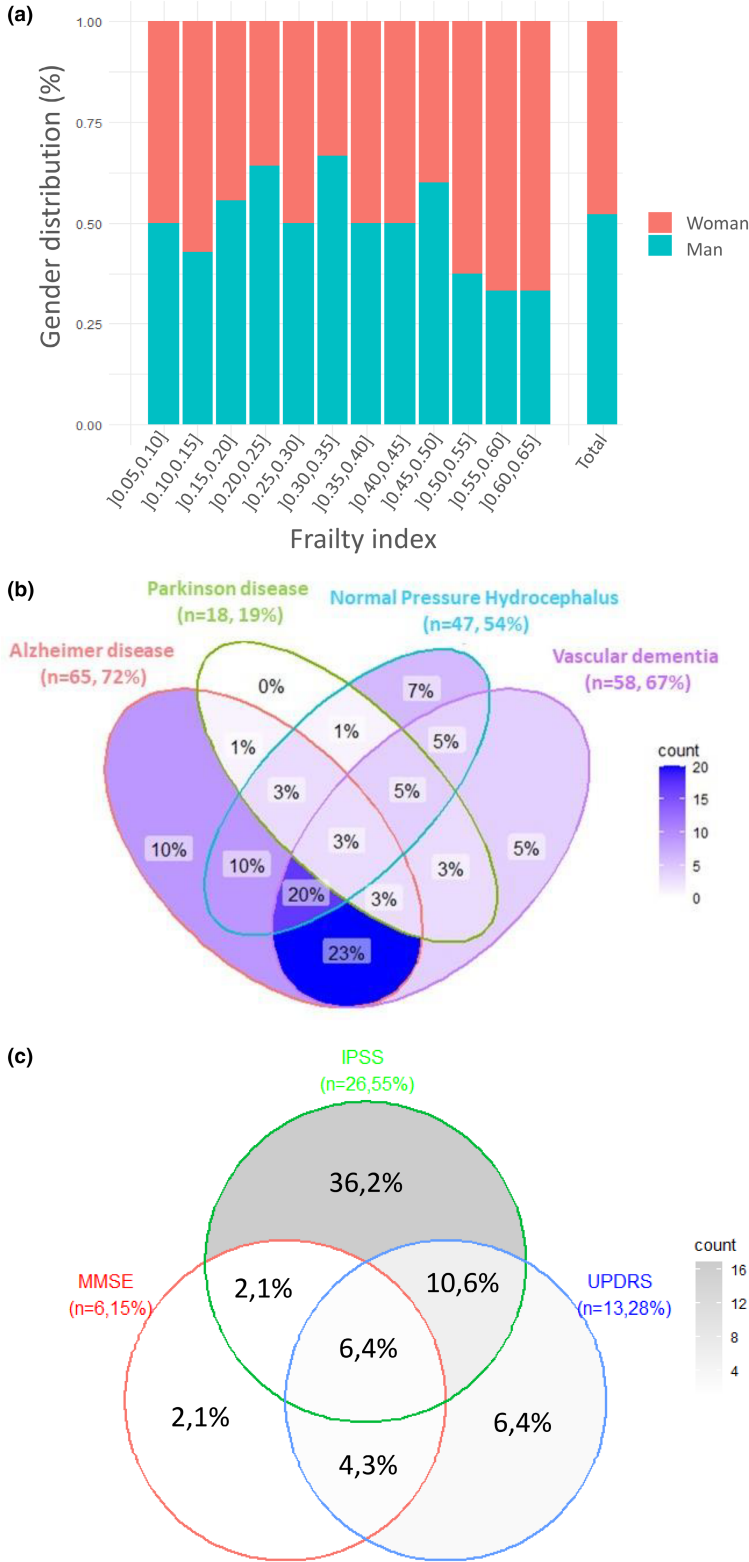
Description of frailty index, biomarkers and clinical parameters of neurodegenerative diseases in our cohort of NPH‐suspected patients. (a) Percentage stacked bar chart of gender distribution by frailty index. (b) Venn diagram showing distribution of subjects of our cohort through markers of Alzheimer disease, Parkinson disease, Normal Pressure Hydrocephalus and Vascular dementia. (c) Venn diagram showing association of clinical scales with the NPH phenotype (*n* = 47). The clinical scales are validated using moderate severity cut‐off values.

After careful evaluation, 10 subjects were excluded from the initial cohort of 100 subjects, for FI reliability criterion or missing data. This resulted in a sample of 90 subjects, with statistical characteristics of demographic variables and FI parameters (Table S2). The construction of the FI does not incorporate enrichment for neurology clinical features. In this cohort, 48% were women. The average subject age was 74.8 years and the average FI was 0.32. The gender distribution based on FI does not reveal a significant predominance of either gender according to the FI values (Figure 1a). Regarding vascular risk factors, 47% were hypertensive, 28% diabetic and 30% had dyslipidemia.

### 3.2 Neurodegenerative profile of the study population

The Venn diagram describes the cohort through the prism of neurodegenerative diseases (Figure 1b). 52% of subjects had *R*_0_ > 12 mmHg/mL/min that is, increased risk of NPH, 72% had a ratio TAU/Aβ42 > 0.215 that is, increased risk of AD, 20% had a presynaptic dopamine deficiency on DATSCAN that is, increased risk of PD, and 69% had a Fazekas scale greater than or equal to grade 2 that is, increased risk of vascular dementia. Only 3% presented no risks of any of the four diseases considered and 33% presented risks of at least three diseases. Herein, we are in the context of a non‐single specific disease cohort. Among the 47 patients exhibiting an NPH phenotype, 55% of patients exceed the moderate severity threshold for the IPSS score, 28% for the UPDRS score and 15% for the MMSE score (Figure 1c). Additionally, 32% of them showed no risks based on any of the three scales.

### 3.3 CSF proteins associated with frailty index

**FIGURE 2:**
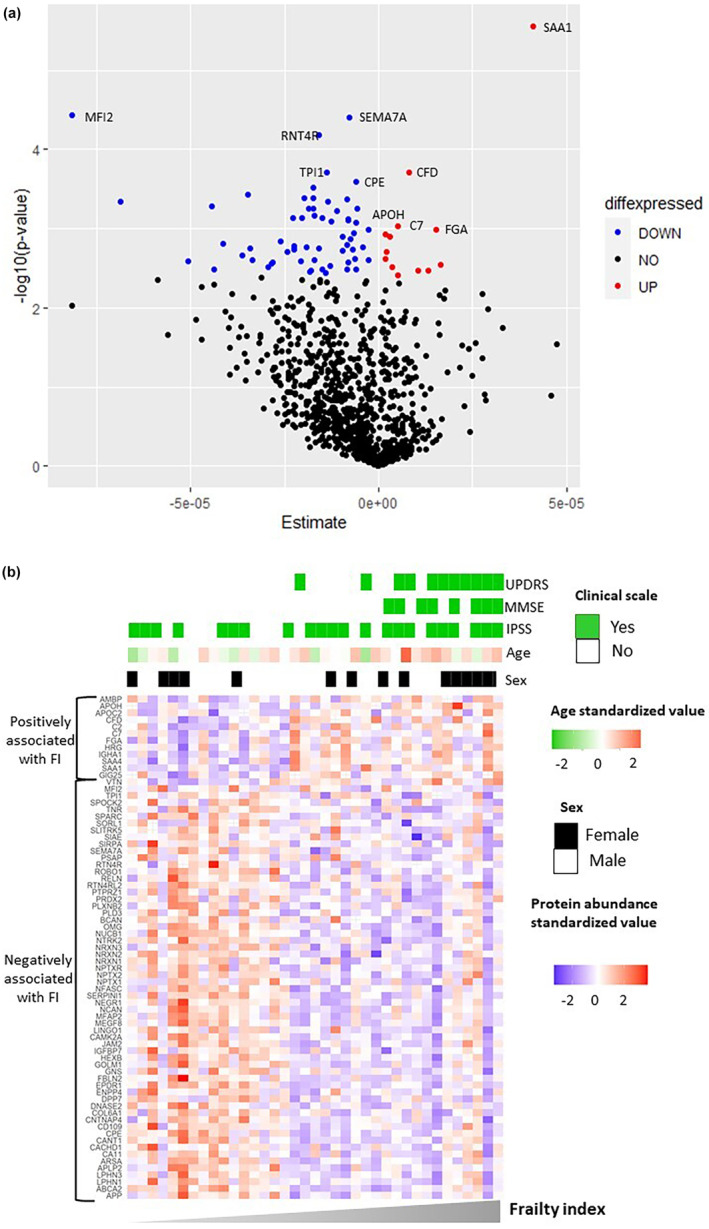
Association of CSF proteins with frailty index. (a) Volcano plot showing associated proteins as red (positively) and blue (negatively) dots (adjusted *p* < 0.05). The *x*‐axis denotes the beta estimate (β1) from the linear model and the *y*‐axis shows the significance level presented as −log10 (*p*‐value). The top five proteins positively and negatively associated proteins with frailty index have been annotated. (b) Heatmap illustrating frailty index‐related changes in CSF proteins based on standardized protein abundance values. The *x*‐axis represents the frailty index, while the *y*‐axis depicts the proteins categorized by their linear association with the FI: negative or positive. The heatmap is supplemented by adjustment factors (age and sex) and clinical scales of the NPH phenotype. The clinical scales are validated using moderate severity cutoff values (yes: above the cutoff; no: below the cutoff).

By using a Pierce technology spin column, depletion of the most abundant proteins to reduce protein dynamic range and high‐throughput mass spectrometry analysis, a total of 1277 proteins could be identified, globally in the samples of the PROLIPHYC cohort. Out of them, 999 proteins quantified in at least 50% of the samples were retained for further statistical analysis. Following data normalization, additional outlier filtering was applied and 932 proteins consistently quantified across the different subjects were further considered. Using Pearson correlation, we analyzed the association between the FI and the 932 proteins abundance, adjusted on age and sex (Table S3). There were 73 proteins that were significantly associated with the cumulative FI (Figure 2a). Of these, 13 proteins were positively associated with the FI, while 60 proteins were negatively associated with the FI. The heatmap illustrated specifically the association between the FI and the 73 significantly associated proteins (Figure 2b). The tables provide various details for each significant protein, including UniProt ID, UniProt accession (protein), full protein name, estimate, standard error, and *p*‐value (Tables S4 and S5). It is similar to the association between the FI and the 932 proteins abundance, adjusted on age, sex, and R_0_ (Figure S1; Tables S6 and S7). We enhanced the heatmap depicting the relationship between protein abundance and FI by incorporating patient descriptions through adjustment factors (age and sex) and clinical scales of the NPH phenotype. Higher values revealed an overrepresentation of women, older patients, and those experiencing neurological dysfunction.

### 3.4 Pathway analysis

**FIGURE 3:**
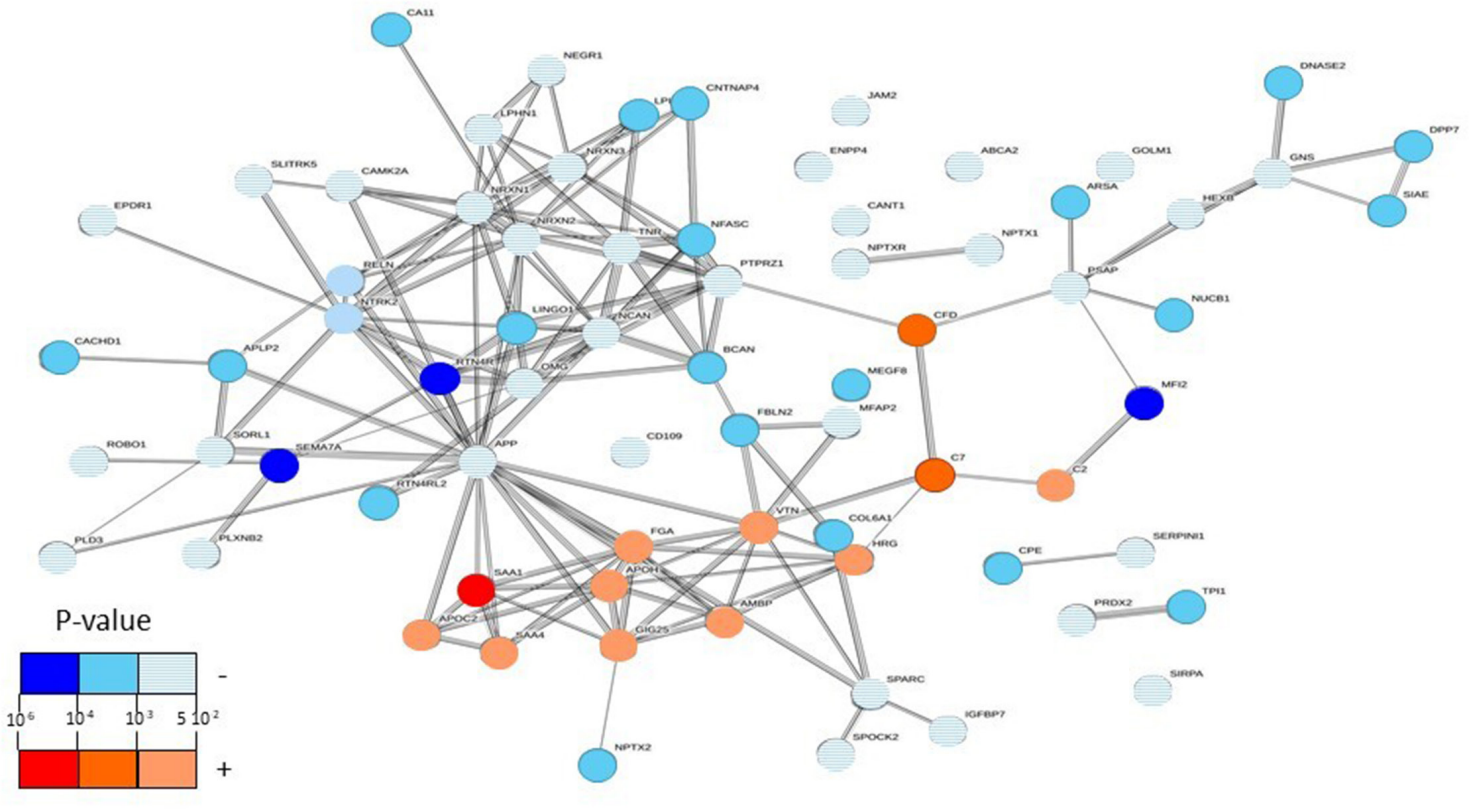
Seventy‐three significant CSF proteins associated with frailty index, illustrated in a proteins network generated by STRING. Blue gradient annotated‐proteins represent the negatively associated proteins. Red gradient annotated‐proteins represent the positively associated proteins. The color gradient selection depends on the *p*‐value magnitude.

Enrichment of functional pathways was then performed on the 73 proteins significantly associated with FI using STRING. The Figure 3 illustrated the associated protein networks.

The top biological process positively associated with FI (red‐annotated proteins) were proteins related to the immune and coagulation pathways. Biological processes positively associated with FI were the regulation of blood coagulation, the platelet degranulation, the acute phase response and the immune response (Table S8). SAA1, CFD, and C7, as the top proteins positively associated with FI, have known roles in such pathways. The results obtained in STRING were confirmed with other tools (GO and Reactome). Indeed, GO analysis showed enrichment of “humoral immune response,” “negative regulation of coagulation,” “defense response,” and “negative regulation of hemostasis” (Table S9). Platelet degranulation and complement cascade were among the top pathways associated with FI in the Reactome pathway analysis (Table S10).

The top biological process negatively associated with FI (blue‐annotated proteins) were proteins related to the central nervous system. Biological processes negatively associated with FI were the regulation of nervous system development, the neurogenesis, the axon development, and the synaptic transmission (Table S11). Numerous proteins are well‐documented in the neurodegenerative disease literature, including SLITRK5, NRXN1, NRXN3, RELN, APP, RTN4R, RTN4RL2, ROBO1, and SEMA7A. The results obtained in STRING were confirmed with other tools (GO and Reactome). GO analysis showed enrichment of “neuron projection development,” “axon development,” “cell adhesion,” and “neuron system development” as top biological processes associated with FI (Table S12). Axon development was among the top pathways associated with FI in the Reactome pathway analysis (Table S13).

### 3.5 Frailty prediction

**FIGURE 4:**
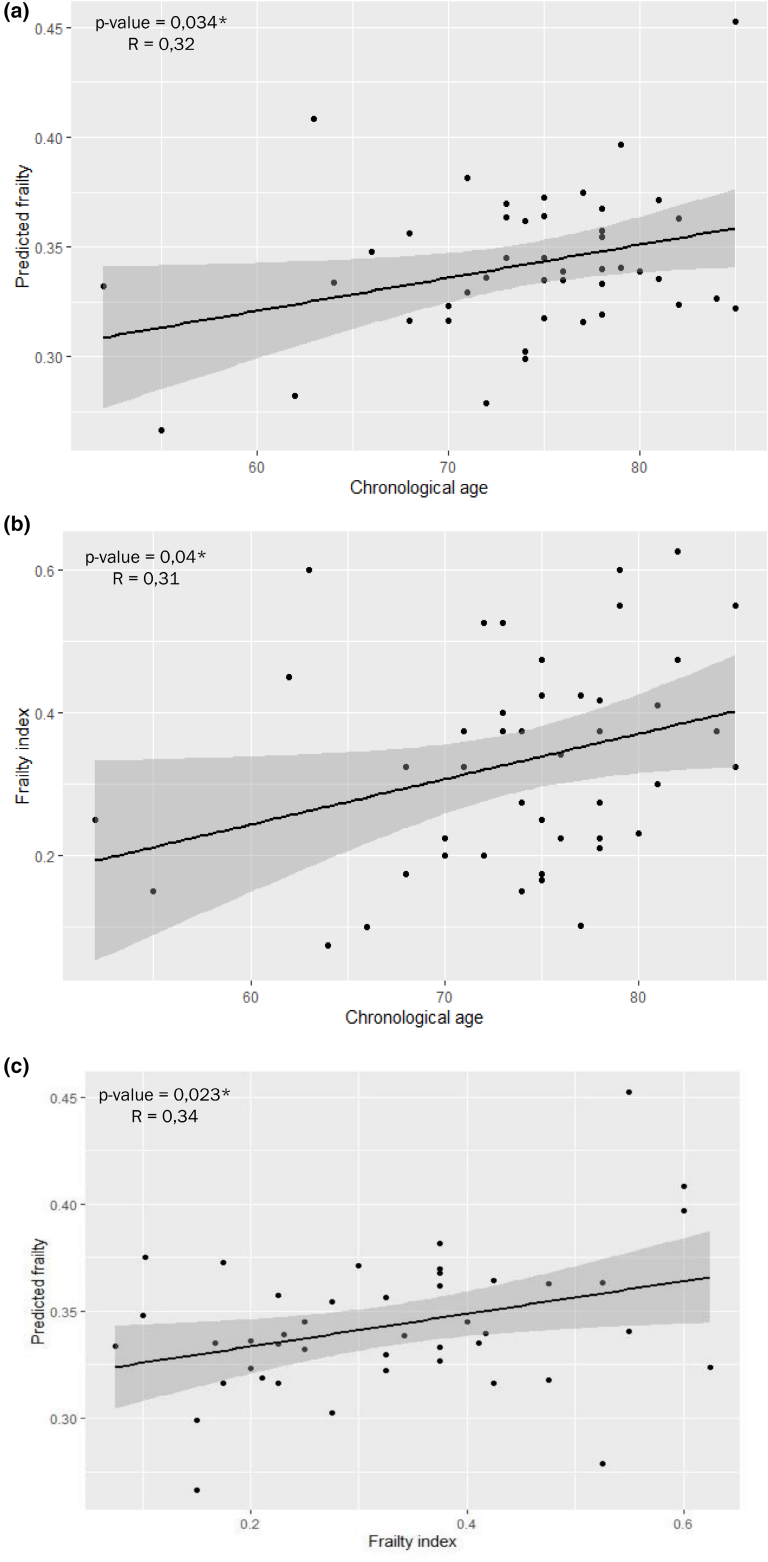
Prediction model of frailty index through proteomic data. Pearson correlation of predicted frailty and chronological age (a), of frailty index and chronological age (b), and of predicted frailty and frailty index (c). * : *p*‐value < 0.05.

In order to obtain a proteomic signature of frailty, we performed a ridge regression with the 932 proteins abundance. Using a stratified random method, we divided our cohort into training and validation sets. Ridge regression was applied to the training set, using all the proteins as estimators to construct a ridge model that predicts the proteomic FI (Table S14). We analyzed the correlation of observed cumulative FI and predicted proteomic FI with chronological age. We found a correlation between chronological age and predicted proteomic FI (*r* = 0.32, *p* = 0.034) (Figure 4a) that is similar to the correlation observed between chronological age and observed FI (*r* = 0.31, *p* = 0.04) (Figure 4b). Additionally, predicted proteomic FI and observed FI are significantly associated (*r* = 0.34, *p* = 0.023) (Figure 4c).

## 4 DISCUSSION

The aim of the present study was to decipher the CSF proteomic signature of frailty. To our knowledge, this is the first quantitative proteomic study to explore the CSF molecular phenotype associated with frailty. Our results revealed a positive association between FI and proteins related to inflammation and immune response. Moreover, most of the 60 proteins negatively associated with frailty are involved in functional pathways linked to neurogenesis, synaptogenesis, and neuronal guidance. Furthermore, we established that frailty predicted by CSF proteomics correlates as consistently with chronological age as it does with FI.

We employed an unbiased approach based on correlation analysis, providing a new methodological perspective compared to the prevalent comparative case–control studies in this field. This approach, which doesn't require a control group, could be seen as more advantageous, as the determination of a suitable control group in frailty studies is challenging.

The statistical analysis of proteomic data is often based on comparison tests. Indeed, most of the studies are case–control studies, where the comparative approach is relevant. This was the case in the first proteomic studies of frailty aiming at the characterization of frailty in subjects, where the statistical approach was based on comparison tests such as the Student's *t*‐test (Lin et al., 2017; Shamsi et al., 2012) and logistic regression (Landino et al., 2021). However, to be relevant, these statistical analyzes require that the subject groups are clinically homogeneous. Obtaining such clinically homogeneous groups of subjects is a challenge in the field of frailty, neurodegenerative diseases and pathological cerebral aging, as the signs of different disorders might often occur simultaneously in a same subject, and a specific diagnosis is difficult to establish.

In recent years, several studies have relied on unbiased approaches to analyze variations in proteomic profiles related to frailty (Sathyan et al., 2020). The control group, essential in comparative trials, is no longer required, as such approaches are essentially based on correlation analysis. Using this correlation approach, we defined a FI and obtained a global, non‐hypothesis driven assessment of the relationship between protein abundances and frailty. An adjustment by age and sex was carried out beforehand, as age is clearly associated with frailty, and previous reports mention a higher prevalence of frailty in women than in men (Fried et al., 2001). The choice of deficits included in the FI was based on the following hypothesis: the prevalence of each deficit increases with chronological age (Rockwood & Mitnitski, 2011).

The identification of 73 proteins associated with frailty provides valuable insights into the molecular mechanisms underlying frailty, with a strong emphasis on the involvement of the immune system and coagulation processes. Particularly, the identification of proteins that were never reported to be associated with frailty in the CNS before brings novel insights that could potentially lead to new therapeutic targets or biomarkers.

Proteins positively associated with FI are known to play a role in coagulation, inflammation and immune response processes. Among them, C7 is a protein encoded by the C7 gene (Chr 5p13.1). It is ubiquitously expressed and is involved in the innate and adaptive immune response by forming pores in the plasma membrane of target cells. Vitronectin is a protein encoded by the VTN gene (Ch 17q11.2). It is mainly expressed in plasma and in the extracellular matrix. VTN is a glycoprotein involved in the complement pathway as a potential chaperone of complement proteins (Menny et al., 2021). Together, these immune markers have been identified as risk factors for AD, as neuron‐derived protein markers of neuronal dysfunction (Zhong et al., 2021). GIG25 is a protein encoded by the SERPINA3 gene (Ch 14q32.13). It is synthesized in liver cells and secreted into the plasma. It plays a role in the acute phase response by stimulating the secretion of cytokines, including IL‐6 and TNFα, through the regulation of the NF‐κB signaling pathway (Jin et al., 2022). It is also a major component of nerve fiber nodules, which play an important role in the pathogenesis of AD (Licastro et al., 1995). As a potential assessor of cognitive decline, it is consistent to find an association between the level of this protein and the frailty phenotype in the CNS. The remaining proteins are part of analogous or identical clusters: acute phase response (AMBP, SAA1 and SAA4), complement activation (C2 and CFD), humoral immune response (IGHA1), blood coagulation (HRG and FGA) and the regulation of lipid metabolism (APOH and APOC2). All of these pathways are known to contribute significantly to frailty.

Another important result of our study is the identification of the role of neuronal plasticity in frailty, revealing associations between frailty and proteins associated with neurodegenerative processes and highlighting potential links between frailty and diseases like AD and PD.

Among the 60 proteins negatively associated with FI, 26 are described to be associated with the development of the nervous system and with specific pathways such as neurogenesis, synaptic organization, and neuronal guidance. First, impairment of neurogenesis was associated with the FI. Among them, SLITRK5 and NTRK2 are known to modulate BDNF‐dependent biological responses through indirect (Song et al., 2015) and direct (Wu et al., 2018) interaction with BDNF, a neuronal growth factor involved in neurogenesis and synaptogenesis.

Second, FI was also associated with variations in the abundance of proteins involved in synaptic plasticity. Indeed, negative correlations were found with different members of the neurexin (NRXN1, NRXN2, NRXN3, and CNTNAP4) and latrophilin (LPHN1 and LPHN3) families, two classes of presynaptic cell adhesion proteins (Chia et al., 2013).

Third, we also found a negative association between FI and certain proteins playing a key role in regulating the interaction between the perineuronal network (PNN), a specialized extracellular matrix occurring around certain populations of neurons. These are TNR, BCAN, and NCAN. BCAN and NCAN are chondroitin sulfate proteoglycans (CSPG) and TNR can cross‐link CSPG in PNN (Mueller‐Buehl et al., 2022). The emergence of PNNs around subsets of neurons is important for synaptic homeostasis. PLXNB2 and SEMA7A, two proteins playing an important role in integrin‐mediated signaling, are negatively associated with FI.

Fourth, we found an association between frailty and several proteins associated with neurodegenerative processes. Indeed, several proteins related to AD were associated with FI, more particularly in amyloid metabolism (Lu et al., 2021; Zhou et al., 2022). We found a negative association between FI and APP, the precursor of β‐amyloid, and its homolog, APLP2, as well as SORL1, a regulator of endosomal recycling of APP (Mishra et al., 2022). In AD, APP undergoes cleavage by β‐secretase, resulting in the production of two amyloid peptides, described as potential CSF biomarkers for this disease (García‐Ayllón et al., 2017). Consequently, accelerated degradation of APP could lead to an increased generation of these two amyloid peptides.

Several studies have already analyzed the plasma proteomic profile of frailty (Landino et al., 2021; Lin et al., 2017; Liu et al., 2023; Mitchell et al., 2023; Sathyan et al., 2020; Shamsi et al., 2012; Verghese et al., 2021). In two small cohorts, studies showed that proteins involved in the inflammatory response and the coagulation pathway differed in frail versus non‐frail subjects (Lin et al., 2017; Shamsi et al., 2012). Another study of a larger cohort (997 subjects) found an association between each protein and frailty status (Landino et al., 2021). The study confirmed that the proteins are involved in inflammation and coagulation. Interestingly, several proteins positively associated with FI in our study have also been identified in previous studies of plasma proteomic analysis in frail subjects (Lin et al., 2017; Liu et al., 2023; Mitchell et al., 2023; Sathyan et al., 2020). Notably, immune response regulators such as C7, GIG25, and VTN were differentially expressed when comparing plasma samples from frail and non‐frail subjects (Lin et al., 2017). Some of proteins negatively associated with FI have also been identified in previous studies of plasma proteomic analysis in frail subjects (Liu et al., 2023; Sathyan et al., 2020; Verghese et al., 2021). These are SEMA7A, BCAN, NCAN, OMG, GOLM1, ROBO1, APLP2, SLITRK5, RTN4R, and LINGO1. They are known to be involved in neuronal guidance (SEMA7A), in the regulation of neuronal interaction (BCAN and NCAN), in neurogenesis (GOLM1, ROBO1, and SLITRK5) and in amyloid pathogenesis (APLP2, RTN4R, OMG, and LINGO1). In the LonGenity cohort, an unbiased approach revealed frailty‐associated proteins related to lipid metabolism, musculoskeletal development, and growth factor pathways (Sathyan et al., 2020; Verghese et al., 2021). More recently, these pathways linked to frailty have been confirmed in two studies involving 1725 elderly people (Liu et al., 2023) and 1044 elderly women (Mitchell et al., 2023).

In the second part of our work, we proposed a proteomic model as an equivalent predictor of FI that could be potentially used in place of clinical and biological criteria. Our model is based on a ridge regression with the 932 proteins abundance. We analyzed the correlation between observed cumulative FI and predicted proteomic FI with chronological age and noted a similar correlation between predicted frailty and chronological age vs FI and chronological age. These results show that the “proteomic model” could become an equivalent predictor of FI in order to study chronological age. Thus, carrying out a proteomic analysis of a biological fluid such as CSF accounts for FI equivalently to the use of a method based on considering 32 clinical criteria, 7 biological criteria, and 1 drug treatment history criteria.

Like the epigenetic clock, a biomarker index that combines information about DNA methylation sites associated with chronological age in multiple tissues and predicts multiple health outcomes, the CSF proteome can serve as a “proteomic clock” (Chen et al., 2016). Here, the predicted frailty suggests that biological aging is associated with molecular changes that can potentially be used to identify individuals who become frail faster or slower. Moreover, this frailty predicted by CSF protein composition could be a good predictor of biological age since it shares similar networks (inflammatory response, complement and coagulation cascades and axonal guidance) with the plasma proteomic signature of chronological age in healthy subjects (Tanaka et al., 2018). Our results suggest that the proteomic analysis of a biological fluid is a promising approach, to be confirmed in larger cohorts, to assess the biological age of different subjects.

The current study has many strengths. Using Pierce technology, we were able to obtain detailed analysis of CSF proteins and low‐level proteins. We have identified and quantified nearly 1000 proteins in these biological samples. To date, this is the only study that has analyzed the proteomics of frailty in CSF. Another strength of the study is the well characterized PROLIPHYC cohort. The participants in the cohort underwent clinical assessments (cognitive, nutritional, and motor) and a validated and reliable cumulative FI deficit was calculated for each subject in order to capture the multidimensional aspects of the frailty phenotype (Guillotin et al., 2022; Vallet et al., 2020). The heterogeneity of the cohort illustrates the diversity of neurodegenerative diseases encountered in elderly subjects: AD, PD, vascular dementia, and NPH. We recognize the limits. The PROLIPHYC cohort is composed of subjects with suspicion of NPH. This suspicion of NPH is the entry point for subjects in the cohort, and allowed us to obtain CSF samples as well as structural and biomechanical data within the framework of care. We have shown in our previous work that the suspicion of NPH does not interfere with the associations identified between biological, biomechanical and clinical parameters (Guillotin et al., 2022; Vallet et al., 2020). It has also recently been shown that ventricular enlargement is a sign of advancing age, not just of suspected NPH (Fujita et al., 2023). Moreover, when we adjust the CSF proteomic profile of frailty for age, sex, and R_0_, it exhibits a striking similarity to the CSF proteomic profile of frailty adjusted for age and sex alone.

Nevertheless, the analysis of this cohort presents certain limitations. Many of these limitations are intrinsic to the cohort itself, including the small sample size, the challenges in generalizing results to the general population, and the inherent limitations of a cross‐sectional design. While the initial cross‐sectional exploration in the PROLIPHYC cohort yields promising findings, future investigations in larger, more diverse populations and longitudinal studies are essential to enhance statistical robustness and deepen our understanding of frailty.

In conclusion, our study has opened up a new avenue for understanding the proteomic signature of frailty, paving the way for further research in this area. Using Pierce technology for a proteomic approach to CSF discovery, we unveiled new associations between proteins and pathways linked to frailty. Our study highlights the unique window provided by the study of the CNS, identifying for the first time the neuroinflammation and neurodegeneration phenomena involved in the pathophysiology of frailty. Beyond simple statistical correlations, it appears that frailty and neurodegenerative diseases share common pathophysiological pathways. Our study also suggests the possibility of building a CSF biological signature that could predict chronological age in the future. In the rapidly advancing field of gerontology, proteomic analysis opens up new perspectives for the biological understanding of the frailty syndrome. If subsequent longitudinal studies demonstrate that changes in this frailty‐related proteomic profile correlate with the phenotypic transformations of frailty, this would not only reveal the biological underpinnings of frailty, but also contribute significantly to the formulation of preventive measures against frailty in our aging population.

## AUTHOR CONTRIBUTIONS

S.G., O.B.S., N.D., and E.S. conceptualized the study design. E.S. acquired the data from the cohort, while A.F., A.G.P., E.M.B., and O.B.S. collected data from the proteomic analysis. S.G., A.G.P., E.M.B. and N.D. analyzed and interpreted the data. S.G., N.D., P.C., S.A., and E.S. contributed to drafting the manuscript. All authors reviewed and approved the final version of the manuscript.

## ACKNOWLEDGMENTS

The authors would like to thank Bernard Monsarrat for initiating the PROLIPHYC project.

## FUNDING INFORMATION

This work was supported by a grant from the Clinical Research Hospital Program from the French Ministry of Health (PHRC 2011‐A01091‐40), by the Occitania Region research funding (RPBIO 2015 no. 14054344), by the European Research Council under the European Union's Seventh Framework Program (FP7/2007‐2013). The work was also funded in part by grants from the Région Occitanie, European funds (Fonds Européens de Développement Régional, FEDER), Toulouse Métropole, and the French Ministry of Research with the Investissement of Avenir Infrastructures Nationales en Biologie et Santé program (ProFI, Proteomics French Infrastructure project, ANR‐10‐INBS‐08).

## CONFLICT OF INTEREST STATEMENT

The authors declare no conflict of interest.

## Supporting information


Data S1.

Data S2.

Data S3.

## Data Availability

The raw MS files and data resulting from the bioinformatics analysis using MaxQuant software can be accessed on the Pride website under the number PXD043497.
