# Supplementary material for: Cerebrospinal fluid proteomic profile of frailty: Results from the PROLIPHYC cohort
Source: Aging Cell. 2024 May 2;23(7):e14168. doi: 10.1111/acel.14168 (PMC11258431; doi:10.1111/acel.14168)
Supplement: Supplementary file 1 — Data S1. [file ACEL-23-e14168-s001.docx]

**Supplementary Table 1:** Variables used in the construction of the frailty index

^a^UPDRS: Unified Parkinson’s Disease Rating Scale / ^b^SEGA: short emergency geriatric assessment / ^c^IPSS: International Prostate Score Symptom / ^d^MNA: Mini Nutritional Assessment

| Number | Parameters | Conditions for deficit |
| --- | --- | --- |
| Executives functions | | |
| 1 | Dependence for dressing | UPDRS^a^ item score >=2 |
| 2 | Dependence for hygiene |  |
| 3 | Handwriting |  |
| 4 | Speech |  |
| 5 | Transferring |  |
| 6 | Chewing and swallowing |  |
| 7 | Doing hobbies and other activities |  |
| Mood and cognition | | |
| 8 | Cognitive impairment | UPDRS^a^ item score >=2 |
| 9 | Depressed mood | SEGA^b^ item > 1 |
| 10 | Apathy | UPDRS^a^ item score >=2 |
| 11 | Self-reported health compared to others | SEGA^b^ item > 1 |
| 12 | Night-time sleep problems | UPDRS^a^ item score >=2 |
| 13 | Anxious mood |  |
| Gait | | |
| 14 | Walking and balance | UPDRS^a^ item score >=2 |
| 15 | Freezing | UPDRS^a^ item score >=2 |
| 16 | Gait |  |
| 17 | Global spontaneity of movement |  |
| Rigidity and agility of extremities | | |
| 18 | Tapping fingers | UPDRS^a^ item score >2 left + right |
| 19 | Tapping toes |  |
| 20 | Rigidity upper extremities |  |
| 21 | Rigidity lower extremities |  |
| 22 | Hand movements |  |
| 23 | Leg agility |  |
| Laboratory blood test | | |
| 24 | Urea blood level | < 2.9 mM or > 8.2 mM |
| 25 | Creatinine blood level | Cockcroft-Gault formula < 60 mL/min/1.73 m^2^ |
| 26 | C-reactive protein blood level | > 3 mg/L |
| 27 | Triglyceride blood level | > 2.25 mM |
| 28 | Glycated hemoglobin | > 5.7% |
| 29 | Cobalamin | < 300 pg/mL |
| 30 | Sodium | < 136 mM or > 142 mM |
| Urinary symptoms | | |
| 31 | Need to urinate every 2 h | IPSS^c^ item >=2 |
| 32 | Nocturia |  |
| 33 | Leakage |  |
| 34 | Sensation of bladder not empty |  |
| Nutrition | | |
| 35 | Body mass index | < 18.5 or > 25 |
| 36 | Loss of appetite | MNA^d^ <=2 |
| 37 | Loss of weight | MNA^d^ <=2 |
| History | | |
| 38 | Drugs history | More than four drugs |
| 39 | Hypertension | Present |
| 40 | Diabetes mellitus | Present |

**Supplementary Table 2:** Clinical characteristics of the PROLIPHYC cohort.

| **Variables** | **PROLIPHYC** |
| --- | --- |
| Participants, n | 90 |
| Age, mean ± SD, years | 74.81 ± 6.59 |
| Women, % | 48 |
| Frailty index, mean ± SD | 0.32 ± 0.14 |
| Comorbidities, %  High blood pressure  Diabetes mellitus  Dyslipidemia | 47  28  30 |

**Supplementary Table 4:** Significant CSF proteins positively associated (increased expression) with frailty index.

| **UniProt** | **Protein** | **Full name protein** | **Estimate** | **Std. Error** | **p-value** |
| --- | --- | --- | --- | --- | --- |
| P0DJI8 | SAA1 | Serum amyloid A-1 protein | 4.10E-05 | 8.07E-06 | 2.74E-06 |
| [P00746](https://www.uniprot.org/uniprot/P00746) | CFD | Complement factor D | 8.24E-06 | 2.11E-06 | 1.94E-04 |
| [P10643](https://www.uniprot.org/uniprot/P10643) | C7 | Complement component C7 | 5.22E-06 | 1.52E-06 | 9.20E-04 |
| [P02671](https://www.uniprot.org/uniprot/P02671) | FGA | Fibrinogen alpha chain | 1.53E-05 | 4.48E-06 | 1.02E-03 |
| [P02749](https://www.uniprot.org/uniprot/P02749) | APOH | Beta-2-glycoprotein 1 | 1.83E-06 | 5.46E-07 | 1.18E-03 |
| [P04004](https://www.uniprot.org/uniprot/P04004) | VTN | Vitronectin | 3.06E-06 | 9.17E-07 | 1.26E-03 |
| [P04196](https://www.uniprot.org/uniprot/P04196) | HRG | Histidine-rich glycoprotein | 1.99E-06 | 6.22E-07 | 1.98E-03 |
| [P01011](https://www.uniprot.org/uniprot/P01011) | GIG25 | Alpha-1-antichymotrypsin | 1.84E-06 | 5.87E-07 | 2.42E-03 |
| [P02655](https://www.uniprot.org/uniprot/P02655) | APOC2 | Apolipoprotein C-II | 1.64E-05 | 5.33E-06 | 2.89E-03 |
| [P02760](https://www.uniprot.org/uniprot/P02760) | AMBP | Protein AMBP | 3.65E-06 | 1.19E-06 | 3.03E-03 |
| [P35542](https://www.uniprot.org/uniprot/P35542) | SAA4 | Serum amyloid A-4 protein | 1.06E-05 | 3.50E-06 | 3.36E-03 |
| [P01876](https://www.uniprot.org/uniprot/P01876) | IGHA1 | Ig alpha-1 chain C region | 1.33E-05 | 4.40E-06 | 3.36E-03 |
| [P06681](https://www.uniprot.org/uniprot/P06681) | C2 | Complement C2 | 5.10E-06 | 1.72E-06 | 3.91E-03 |

**Supplementary Table 5:** Significant CSF proteins associated negatively (decreased expression) with frailty index.

| **UniProt** | **Protein** | **Full name protein** | **Estimate** | **Std. Error** | **p-value** |
| --- | --- | --- | --- | --- | --- |
| P08582 | MFI2 | Melanotransferrin | -8,15E-05 | 1,86E-05 | 3,72E-05 |
| [O75326](https://www.uniprot.org/uniprot/O75326) | SEMA7A | Semaphorin-7A | -7,65E-06 | 1,76E-06 | 3,93E-05 |
| [Q9BZR6](https://www.uniprot.org/uniprot/Q9BZR6) | RTN4R | Reticulon-4 | -1,59E-05 | 3,78E-06 | 6,59E-05 |
| [P60174](https://www.uniprot.org/uniprot/P60174) | TPI1 | Triosephosphate isomerase | -1,39E-05 | 3,57E-06 | 1,95E-04 |
| [P16870](https://www.uniprot.org/uniprot/P16870) | CPE | Carboxypeptidase E | -6,03E-06 | 1,58E-06 | 2,55E-04 |
| [Q9HAT2](https://www.uniprot.org/uniprot/Q9HAT2) | SIAE | Sialate O-acetylesterase | -1,73E-05 | 4,58E-06 | 2,99E-04 |
| [O00115](https://www.uniprot.org/uniprot/O00115) | DNASE2 | Deoxyribonuclease-2-alpha | -3,48E-05 | 9,37E-06 | 3,68E-04 |
| [Q5VU97](https://www.uniprot.org/uniprot/Q5VU97) | CACHD1 | VWFA and cache domain-containing protein 1 | -1,98E-05 | 5,39E-06 | 4,07E-04 |
| [P98095](https://www.uniprot.org/uniprot/P98095) | FBLN2 | Fibulin-2 | -1,75E-05 | 4,75E-06 | 4,14E-04 |
| [P12109](https://www.uniprot.org/uniprot/P12109) | COL6A1 | Collagen alpha-1(VI) chain | -8,30E-06 | 2,26E-06 | 4,29E-04 |
| [Q9C0A0](https://www.uniprot.org/uniprot/Q9C0A0) | CNTNAP4 | Contactin-associated protein-like 4 | -1,35E-05 | 3,69E-06 | 4,52E-04 |
| [O75493](https://www.uniprot.org/uniprot/O75493) | CAH11 | Carbonic anhydrase-related protein 11 | -6,88E-05 | 1,88E-05 | 4,58E-04 |
| [P15289](https://www.uniprot.org/uniprot/P15289) | ARSA | Arylsulfatase A | -4,45E-05 | 1,23E-05 | 5,18E-04 |
| [Q02818](https://www.uniprot.org/uniprot/Q02818) | NUCB1 | Nucleobindin-1 | -1,86E-05 | 5,19E-06 | 5,58E-04 |
| [Q96FE5](https://www.uniprot.org/uniprot/Q96FE5) | LINGO1 | Leucine-rich repeat and immunoglobulin-like domain-containing nogo receptor-interacting protein | -1,75E-05 | 4,88E-06 | 5,60E-04 |
| [Q96GW7](https://www.uniprot.org/uniprot/Q96GW7) | BCAN | Brevican core protein | -5,73E-06 | 1,60E-06 | 5,67E-04 |
| [O94856](https://www.uniprot.org/uniprot/O94856) | NFASC | Neurofascin | -1,10E-05 | 3,08E-06 | 5,89E-04 |
| [Q86UN3](https://www.uniprot.org/uniprot/Q86UN3) | RTN4RL2 | Reticulon-4 receptor-like 2 | -1,72E-05 | 4,87E-06 | 6,77E-04 |
| [Q9UHL4](https://www.uniprot.org/uniprot/Q9UHL4) | DPP7 | Dipeptidyl peptidase 2 | -1,49E-05 | 4,24E-06 | 7,35E-04 |
| [P47972](https://www.uniprot.org/uniprot/P47972) | NPTX2 | Neuronal pentraxin-2 | -2,04E-05 | 5,81E-06 | 7,41E-04 |
| [Q9HAR2](https://www.uniprot.org/uniprot/Q9HAR2) | LPHN3 | Adhesion G protein-coupled receptor L3 | -2,29E-05 | 6,55E-06 | 7,43E-04 |
| [Q06481](https://www.uniprot.org/uniprot/Q06481) | APLP2 | Amyloid beta precursor like protein 2 | -8,12E-06 | 2,32E-06 | 7,69E-04 |
| [Q7Z7M0](https://www.uniprot.org/uniprot/Q7Z7M0) | MEGF8 | Multiple epidermal growth factor-like domains protein 8 | -8,05E-06 | 2,31E-06 | 7,90E-04 |
| [P23471](https://www.uniprot.org/uniprot/P23471) | PTPRZ1 | Receptor-type tyrosine-protein phosphatase zeta | -1,25E-05 | 3,58E-06 | 8,08E-04 |
| [P07602](https://www.uniprot.org/uniprot/P07602) | PSAP | Prosaposin | -6,13E-06 | 1,77E-06 | 8,53E-04 |
| [Q16270](https://www.uniprot.org/uniprot/Q16270) | IGFBP7 | Insulin-like growth factor-binding protein 7 | -2,72E-06 | 7,99E-07 | 1,04E-03 |
| [O95502](https://www.uniprot.org/uniprot/O95502) | NPTXR | Neuronal pentraxin receptor | -6,45E-06 | 1,91E-06 | 1,14E-03 |
| [Q9P2S2](https://www.uniprot.org/uniprot/Q9P2S2) | NRXN2 | Neurexin-2 | -9,49E-06 | 2,85E-06 | 1,27E-03 |
| [Q7Z3B1](https://www.uniprot.org/uniprot/Q7Z3B1) | NEGR1 | Neuronal growth regulator 1 | -7,54E-06 | 2,27E-06 | 1,35E-03 |
|  |  |  |  |  |  |
| [P32119](https://www.uniprot.org/uniprot/P32119) | PRDX2 | Peroxiredoxin-2 | -2,62E-05 | 7,94E-06 | 1,44E-03 |
| [Q9UM22](https://www.uniprot.org/uniprot/Q9UM22) | EPDR1 | Mammalian ependymin-related protein 1 | -4,13E-05 | 1,26E-05 | 1,57E-03 |
| [O14594](https://www.uniprot.org/uniprot/O14594) | NCAN | Neurocan core protein | -8,54E-06 | 2,61E-06 | 1,58E-03 |
| [P07686](https://www.uniprot.org/uniprot/P07686) | HEXB | Beta-hexosaminidase subunit beta | -2,25E-05 | 6,91E-06 | 1,67E-03 |
| [P78324](https://www.uniprot.org/uniprot/P78324) | SIRPA | Tyrosine-protein phosphatase non-receptor type substrate 1 | -4,19E-06 | 1,29E-06 | 1,73E-03 |
| [Q8NBJ4](https://www.uniprot.org/uniprot/Q8NBJ4) | GOLM1 | Golgi membrane protein 1 | -1,91E-05 | 5,91E-06 | 1,74E-03 |
| [Q9Y6N7](https://www.uniprot.org/uniprot/Q9Y6N7) | ROBO1 | Roundabout homolog 1 | -1,60E-05 | 4,96E-06 | 1,79E-03 |
| [P55001](https://www.uniprot.org/uniprot/P55001) | MFAP2 | Microfibrillar-associated protein 2 | -3,42E-05 | 1,06E-05 | 1,80E-03 |
| [Q9UQM7](https://www.uniprot.org/uniprot/Q9UQM7) | CAMK2A | Calcium/calmodulin-dependent protein kinase type II subunit alpha | -2,24E-05 | 6,94E-06 | 1,82E-03 |
| [P09486](https://www.uniprot.org/uniprot/P09486) | SPARC | SPARC | -6,85E-06 | 2,13E-06 | 1,86E-03 |
| [P23515](https://www.uniprot.org/uniprot/P23515) | OMG | Oligodendrocyte-myelin glycoprotein | -9,46E-06 | 2,95E-06 | 1,92E-03 |
| [Q8WVQ1](https://www.uniprot.org/uniprot/Q8WVQ1) | CANT1 | Soluble calcium-activated nucleotidase 1 | -2,43E-05 | 7,59E-06 | 1,95E-03 |
| [O94991](https://www.uniprot.org/uniprot/O94991) | SLITRK5 | SLIT and NTRK-like protein 5 | -3,64E-05 | 1,15E-05 | 2,14E-03 |
| [P78509](https://www.uniprot.org/uniprot/P78509) | RELN | Reelin | -6,37E-06 | 2,03E-06 | 2,40E-03 |
| [O94910](https://www.uniprot.org/uniprot/O94910) | LPHN1 | Adhesion G protein-coupled receptor L1 | -1,71E-05 | 5,47E-06 | 2,47E-03 |
| [P15586](https://www.uniprot.org/uniprot/P15586) | GNS | N-acetylglucosamine-6-sulfatase | -3,36E-05 | 1,08E-05 | 2,50E-03 |
| [P05067](https://www.uniprot.org/uniprot/P05067) | APP | Amyloid-beta precursor protein | -2,59E-06 | 8,32E-07 | 2,53E-03 |
| [Q9Y6X5](https://www.uniprot.org/uniprot/Q9Y6X5) | ENPP4 | Bis(5-adenosyl)-triphosphatase | -5,08E-05 | 1,63E-05 | 2,54E-03 |
| [Q16620](https://www.uniprot.org/uniprot/Q16620) | NTRK2 | BDNF/NT-3 growth factors receptor | -2,06E-05 | 6,63E-06 | 2,62E-03 |
| [Q9Y4C0](https://www.uniprot.org/uniprot/Q9Y4C0) | NRXN3 | Neurexin-3 | -8,04E-06 | 2,60E-06 | 2,68E-03 |
| [Q8IV08](https://www.uniprot.org/uniprot/Q8IV08) | PLD3 | Phospholipase D3 | -2,83E-05 | 9,15E-06 | 2,71E-03 |
| [Q6YHK3](https://www.uniprot.org/uniprot/Q6YHK3) | CD109 | CD109 antigen | -2,85E-05 | 9,24E-06 | 2,79E-03 |
| [Q99574](https://www.uniprot.org/uniprot/Q99574) | SERPINI1 | Neuroserpin | -1,28E-05 | 4,16E-06 | 2,92E-03 |
| [Q9BZC7](https://www.uniprot.org/uniprot/Q9BZC7) | ABCA2 | ATP-binding cassette sub-family A member 2 | -2,94E-05 | 9,65E-06 | 3,07E-03 |
| [Q9ULB1](https://www.uniprot.org/uniprot/Q9ULB1) | NRXN1 | Neurexin-1 | -8,37E-06 | 2,76E-06 | 3,24E-03 |
| [P57087](https://www.uniprot.org/uniprot/P57087) | JAM2 | Junctional adhesion molecule B | -4,39E-05 | 1,45E-05 | 3,24E-03 |
| [Q15818](https://www.uniprot.org/uniprot/Q15818) | NPTX1 | Neuronal pentraxin-1 | -6,05E-06 | 1,99E-06 | 3,25E-03 |
| [Q92673](https://www.uniprot.org/uniprot/Q92673) | SORL1 | Sortilin-related receptor | -1,51E-05 | 4,98E-06 | 3,28E-03 |
| [O15031](https://www.uniprot.org/uniprot/O15031) | PLXNB2 | Plexin-B2 | -1,79E-05 | 5,93E-06 | 3,34E-03 |
| [Q92752](https://www.uniprot.org/uniprot/Q92752) | TNR | Tenascin-R | -1,82E-05 | 6,08E-06 | 3,54E-03 |
| [Q92563](https://www.uniprot.org/uniprot/Q92563) | SPOCK2 | Testican-2 | -1,42E-05 | 4,75E-06 | 3,65E-03 |


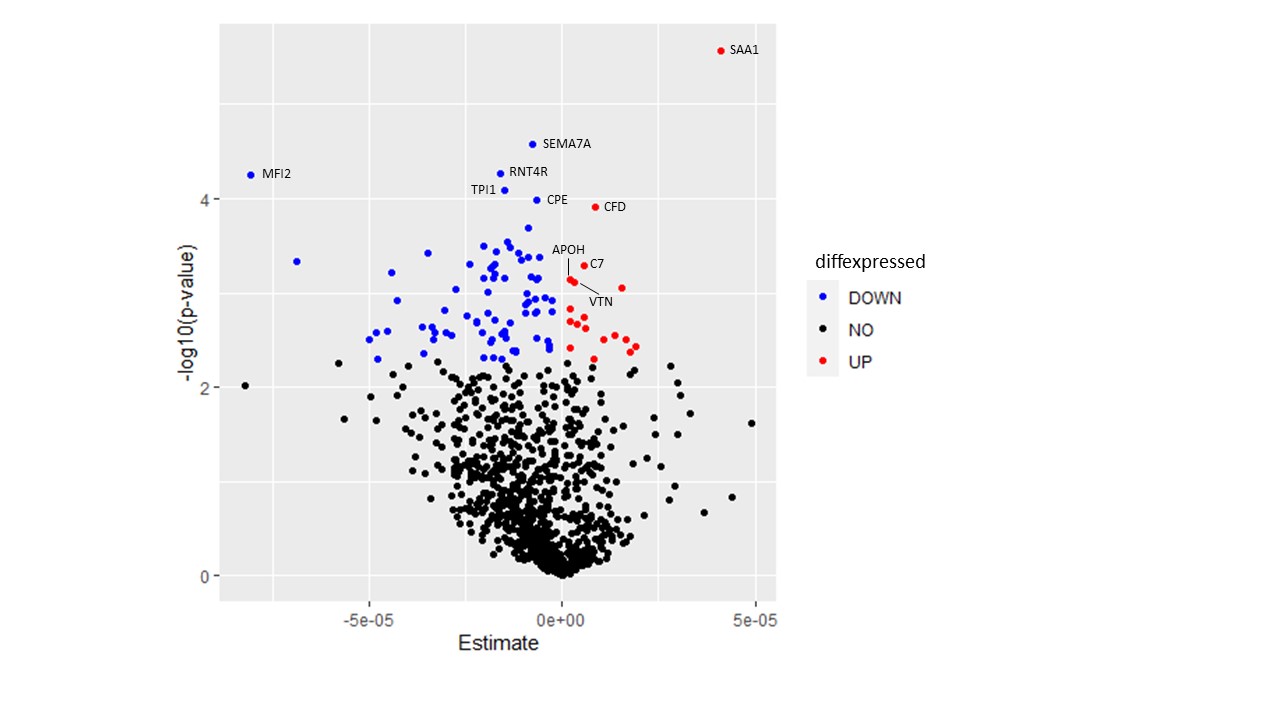


**Supplementary Figure 1:** Association of proteins with frailty index, adjusted on age, sex and R0. Volcano plot showing associated proteins as red (positively) and blue (negatively) dots (adjusted p-value < 0.05). The X-axis denotes the beta estimate (β1) from the linear model and the Y-axis shows the significance level presented as -log10(p-value). The top five proteins positively and negatively associated proteins with frailty index have been annotated.

**Supplementary Table 6:** Significant CSF proteins positively associated (increased expression) with frailty index. Association adjusted on age, sex and R0.

| **UniProt** | **Protein** | **Full name protein** | **Estimate** | **Std. Error** | **p-value** |
| --- | --- | --- | --- | --- | --- |
| P0DJI8 | SAA1 | Serum amyloid A-1 protein | 4,13E-05 | 8,11E-06 | 2,67E-06 |
| [P00746](https://www.uniprot.org/uniprot/P00746) | CFD | Complement factor D | 8,57E-06 | 2,13E-06 | 1,22E-04 |
| [P10643](https://www.uniprot.org/uniprot/P10643) | C7 | Complement component C7 | 5,55E-06 | 1,54E-06 | 5,19E-04 |
| [P02749](https://www.uniprot.org/uniprot/P02749) | APOH | Beta-2-glycoprotein 1 | 1,94E-06 | 5,51E-07 | 7,16E-04 |
| [P04004](https://www.uniprot.org/uniprot/P04004) | VTN | Vitronectin | 3,23E-06 | 9,26E-07 | 7,78E-04 |
| [P02671](https://www.uniprot.org/uniprot/P02671) | FGA | Fibrinogen alpha chain | 1,55E-05 | 4,49E-06 | 8,75E-04 |
| [P04196](https://www.uniprot.org/uniprot/P04196) | HRG | Histidine-rich glycoprotein | 2,06E-06 | 6,26E-07 | 1,47E-03 |
| [P06681](https://www.uniprot.org/uniprot/P06681) | C2 | Complement C2 | 5,68E-06 | 1,76E-06 | 1,81E-03 |
| [P01011](https://www.uniprot.org/uniprot/P01011) | GIG25 | Alpha-1-antichymotrypsin | 1,88E-06 | 5,88E-07 | 2,02E-03 |
| [P02760](https://www.uniprot.org/uniprot/P02760) | AMBP | Protein AMBP | 3,82E-06 | 1,20E-06 | 2,12E-03 |
| [P05543](https://www.uniprot.org/uniprotkb/P05543/entry) | THBG | Thyroxine-binding globulin | 6,04E-06 | 1,92E-06 | 2,37E-03 |
| [P01876](https://www.uniprot.org/uniprot/P01876) | IGHA1 | Ig alpha-1 chain C region | 1,36E-05 | 4,41E-06 | 2,78E-03 |
| [P02655](https://www.uniprot.org/uniprot/P02655) | APOC2 | Apolipoprotein C-II | 1,63E-05 | 5,36E-06 | 3,08E-03 |
| [P35542](https://www.uniprot.org/uniprot/P35542) | SAA4 | Serum amyloid A-4 protein | 1,07E-05 | 3,52E-06 | 3,17E-03 |
| [Q9NZP8](https://www.uniprot.org/uniprotkb/Q9NZP8/entry) | C1RL | Complement C1r subcomponent-like protein | 1,92E-05 | 6,41E-06 | 3,65E-03 |
| [P02652](https://www.uniprot.org/uniprotkb/P02652/entry) | APOA2 | Apolipoprotein A-II | 2,00E-06 | 6,71E-07 | 3,78E-03 |
| [O14791](https://www.uniprot.org/uniprotkb/O14791/entry) | APOL1 | Apolipoprotein L1 | 1,76E-05 | 5,97E-06 | 4,21E-03 |
| [Q6UXB8](https://www.uniprot.org/uniprotkb/Q6UXB8/entry) | PI16 | Peptidase inhibitor 16 | 8,05E-06 | 2,80E-06 | 5,10E-03 |

**Supplementary Table 7:** Significant CSF proteins negatively associated (decreased expression) with frailty index. Association adjusted on age, sex and R0.

| **UniProt** | **Protein** | **Full name protein** | **Estimate** | **Std. Error** | **p-value** |
| --- | --- | --- | --- | --- | --- |
| [O75326](https://www.uniprot.org/uniprot/O75326) | SEMA7A | Semaphorin-7A | -7,87E-06 | 1,77E-06 | 2,66E-05 |
| [Q9BZR6](https://www.uniprot.org/uniprot/Q9BZR6) | RTN4R | Reticulon-4 | -1,61E-05 | 3,79E-06 | 5,49E-05 |
| P08582 | MFI2 | Melanotransferrin | -8,06E-05 | 1,89E-05 | 5,52E-05 |
| [P60174](https://www.uniprot.org/uniprot/P60174) | TPI1 | Triosephosphate isomerase | -1,50E-05 | 3,62E-06 | 8,12E-05 |
| [P16870](https://www.uniprot.org/uniprot/P16870) | CPE | Carboxypeptidase E | -6,53E-06 | 1,60E-06 | 1,05E-04 |
| [P12109](https://www.uniprot.org/uniprot/P12109) | COL6A1 | Collagen alpha-1(VI) chain | -8,92E-06 | 2,29E-06 | 2,02E-04 |
| [Q9C0A0](https://www.uniprot.org/uniprot/Q9C0A0) | CNTNAP4 | Contactin-associated protein-like 4 | -1,41E-05 | 3,71E-06 | 2,90E-04 |
| [Q5VU97](https://www.uniprot.org/uniprot/Q5VU97) | CACHD1 | VWFA and cache domain-containing protein 1 | -2,03E-05 | 5,41E-06 | 3,18E-04 |
| [P23471](https://www.uniprot.org/uniprot/P23471) | PTPRZ1 | Receptor-type tyrosine-protein phosphatase zeta | -1,37E-05 | 3,65E-06 | 3,32E-04 |
|  | SIAE |  | -1,72E-05 | 4,62E-06 | 3,63E-04 |
| [O00115](https://www.uniprot.org/uniprot/O00115) | DNASE2 | Deoxyribonuclease-2-alpha | -3,49E-05 | 9,40E-06 | 3,83E-04 |
| [O94856](https://www.uniprot.org/uniprot/O94856) | NFASC | Neurofascin | -1,15E-05 | 3,10E-06 | 3,83E-04 |
| [Q96GW7](https://www.uniprot.org/uniprot/Q96GW7) | BCAN | Brevican core protein | -5,91E-06 | 1,61E-06 | 4,14E-04 |
| [Q06481](https://www.uniprot.org/uniprot/Q06481) | APLP2 | Amyloid beta precursor like protein 2 | -8,65E-06 | 2,35E-06 | 4,15E-04 |
| [Q9P2S2](https://www.uniprot.org/uniprot/Q9P2S2) | NRXN2 | Neurexin-2 | -1,07E-05 | 2,92E-06 | 4,48E-04 |
| [O75493](https://www.uniprot.org/uniprot/O75493) | CAH11 | Carbonic anhydrase-related protein 11 | -6,89E-05 | 1,89E-05 | 4,62E-04 |
| [Q9HAR2](https://www.uniprot.org/uniprot/Q9HAR2) | LPHN3 | Adhesion G protein-coupled receptor L3 | -2,39E-05 | 6,60E-06 | 4,93E-04 |
| [P98095](https://www.uniprot.org/uniprot/P98095) | FBLN2 | Fibulin-2 | -1,73E-05 | 4,78E-06 | 4,93E-04 |
| [Q96FE5](https://www.uniprot.org/uniprot/Q96FE5) | LINGO1 | Leucine-rich repeat and immunoglobulin-like domain-containing nogo receptor-interacting protein | -1,77E-05 | 4,89E-06 | 5,14E-04 |
| [Q02818](https://www.uniprot.org/uniprot/Q02818) | NUCB1 | Nucleobindin-1 | -1,87E-05 | 5,21E-06 | 5,54E-04 |
| [P15289](https://www.uniprot.org/uniprot/P15289) | ARSA | Arylsulfatase A | -4,42E-05 | 1,24E-05 | 6,07E-04 |
| [Q86UN3](https://www.uniprot.org/uniprot/Q86UN3) | RTN4RL2 | Reticulon-4 receptor-like 2 | -1,74E-05 | 4,89E-06 | 6,30E-04 |
| [Q7Z7M0](https://www.uniprot.org/uniprot/Q7Z7M0) | MEGF8 | Multiple epidermal growth factor-like domains protein 8 | -8,21E-06 | 2,32E-06 | 6,65E-04 |
| [Q7Z3B1](https://www.uniprot.org/uniprot/Q7Z3B1) | NEGR1 | Neuronal growth regulator 1 | -8,16E-06 | 2,31E-06 | 6,66E-04 |
| [P47972](https://www.uniprot.org/uniprot/P47972) | NPTX2 | Neuronal pentraxin-2 | -2,05E-05 | 5,83E-06 | 6,99E-04 |
| [Q9Y6N7](https://www.uniprot.org/uniprot/Q9Y6N7) | ROBO1 | Roundabout homolog 1 | -1,79E-05 | 5,08E-06 | 7,01E-04 |
| [Q9UHL4](https://www.uniprot.org/uniprot/Q9UHL4) | DPP7 | Dipeptidyl peptidase 2 | -1,50E-05 | 4,25E-06 | 7,01E-04 |
| [P07602](https://www.uniprot.org/uniprot/P07602) | PSAP | Prosaposin | -6,26E-06 | 1,78E-06 | 7,02E-04 |
| [O95502](https://www.uniprot.org/uniprot/O95502) | NPTXR | Neuronal pentraxin receptor | -6,78E-06 | 1,93E-06 | 7,21E-04 |
| [P32119](https://www.uniprot.org/uniprot/P32119) | PRDX2 | Peroxiredoxin-2 | -2,78E-05 | 8,06E-06 | 9,17E-04 |
| [O94910](https://www.uniprot.org/uniprot/O94910) | LPHN1 | Adhesion G protein-coupled receptor L1 | -1,91E-05 | 5,61E-06 | 9,93E-04 |
| [O14594](https://www.uniprot.org/uniprot/O14594) | NCAN | Neurocan core protein | -9,01E-06 | 2,64E-06 | 1,00E-03 |
| [P78324](https://www.uniprot.org/uniprot/P78324) | SIRPA | Tyrosine-protein phosphatase non-receptor type substrate 1 | -4,41E-06 | 1,30E-06 | 1,11E-03 |
| [P78509](https://www.uniprot.org/uniprot/P78509) | RELN | Reelin | -6,97E-06 | 2,07E-06 | 1,17E-03 |
| [Q9UM22](https://www.uniprot.org/uniprot/Q9UM22) | EPDR1 | Mammalian ependymin-related protein 1 | -4,26E-05 | 1,27E-05 | 1,19E-03 |
| [Q16270](https://www.uniprot.org/uniprot/Q16270) | IGFBP7 | Insulin-like growth factor-binding protein 7 | -2,70E-06 | 8,05E-07 | 1,22E-03 |
| [Q9Y4C0](https://www.uniprot.org/uniprot/Q9Y4C0) | NRXN3 | Neurexin-3 | -8,87E-06 | 2,65E-06 | 1,24E-03 |
| [Q9ULB1](https://www.uniprot.org/uniprot/Q9ULB1) | NRXN1 | Neurexin-1 | -9,40E-06 | 2,83E-06 | 1,34E-03 |
| [Q8IV08](https://www.uniprot.org/uniprot/Q8IV08) | PLD3 | Phospholipase D3 | -3,04E-05 | 9,27E-06 | 1,55E-03 |
| [P05067](https://www.uniprot.org/uniprot/P05067) | APP | Amyloid-beta precursor protein | -2,75E-06 | 8,41E-07 | 1,56E-03 |
| [Q15818](https://www.uniprot.org/uniprot/Q15818) | NPTX1 | Neuronal pentraxin-1 | -6,64E-06 | 2,03E-06 | 1,60E-03 |
| [P23515](https://www.uniprot.org/uniprot/P23515) | OMG | Oligodendrocyte-myelin glycoprotein | -9,67E-06 | 2,96E-06 | 1,61E-03 |
| [Q8NBJ4](https://www.uniprot.org/uniprot/Q8NBJ4) | GOLM1 | Golgi membrane protein 1 | -1,93E-05 | 5,93E-06 | 1,62E-03 |
| [P09486](https://www.uniprot.org/uniprot/P09486) | SPARC | SPARC | -6,95E-06 | 2,14E-06 | 1,66E-03 |
| [Q8WVQ1](https://www.uniprot.org/uniprot/Q8WVQ1) | CANT1 | Soluble calcium-activated nucleotidase 1 | -2,46E-05 | 7,62E-06 | 1,76E-03 |
| [O60241](https://www.uniprot.org/uniprotkb/O60241/entry) | AGRB2 | Adhesion G protein-coupled receptor B2 | -1,76E-05 | 5,50E-06 | 1,91E-03 |
| [P07686](https://www.uniprot.org/uniprot/P07686) | HEXB | Beta-hexosaminidase subunit beta | -2,22E-05 | 6,97E-06 | 2,02E-03 |
| [Q99574](https://www.uniprot.org/uniprot/Q99574) | NEUS | Neuroserpin | -1,33E-05 | 4,19E-06 | 2,07E-03 |
| [Q9UQM7](https://www.uniprot.org/uniprot/Q9UQM7) | CAMK2A | Calcium/calmodulin-dependent protein kinase type II subunit alpha | -2,22E-05 | 6,98E-06 | 2,08E-03 |
| [O94991](https://www.uniprot.org/uniprot/O94991) | SLITRK5 | SLIT and NTRK-like protein 5 | -3,63E-05 | 1,15E-05 | 2,27E-03 |
| [P55001](https://www.uniprot.org/uniprot/P55001) | MFAP2 | Microfibrillar-associated protein 2 | -3,39E-05 | 1,08E-05 | 2,32E-03 |
| [Q92563](https://www.uniprot.org/uniprot/Q92563) | SPOCK2 | Testican-2 | -1,49E-05 | 4,79E-06 | 2,53E-03 |
| [P57087](https://www.uniprot.org/uniprot/P57087) | JAM2 | Junctional adhesion molecule B | -4,53E-05 | 1,46E-05 | 2,56E-03 |
| [O14917](https://www.uniprot.org/uniprotkb/O14917/entry) | PCD17 | Protocadherin-17 | -3,29E-05 | 1,06E-05 | 2,60E-03 |
| [Q9BZC7](https://www.uniprot.org/uniprot/Q9BZC7) | ABCA2 | ATP-binding cassette sub-family A member 2 | -3,01E-05 | 9,69E-06 | 2,61E-03 |
| [Q16620](https://www.uniprot.org/uniprot/Q16620) | NTRK2 | BDNF/NT-3 growth factors receptor | -2,06E-05 | 6,65E-06 | 2,66E-03 |
| [Q99946](https://www.uniprot.org/uniprotkb/Q99946/entry) | PRRT1 | Proline-rich transmembrane protein 1 | -4,81E-05 | 1,55E-05 | 2,67E-03 |
| [Q9NT99](https://www.uniprot.org/uniprotkb/Q9NT99/entry) | LRC4B | Leucine-rich repeat-containing protein 4B | -1,48E-05 | 4,79E-06 | 2,70E-03 |
| [Q92673](https://www.uniprot.org/uniprot/Q92673) | SORL1 | Sortilin-related receptor | -1,55E-05 | 5,00E-06 | 2,70E-03 |
| [Q6YHK3](https://www.uniprot.org/uniprot/Q6YHK3) | CD109 | CD109 antigen | -2,86E-05 | 9,27E-06 | 2,78E-03 |
| [Q9P121](https://www.uniprot.org/uniprotkb/Q9P121/entry) | NTRI | Neurotrimin | -6,49E-06 | 2,12E-06 | 2,98E-03 |
| [P09104](https://www.uniprot.org/uniprotkb/P09104/entry) | ENOG | Gamma-enolase | -1,47E-05 | 4,81E-06 | 3,06E-03 |
| [O15031](https://www.uniprot.org/uniprot/O15031) | PLXNB2 | Plexin-B2 | -1,81E-05 | 5,95E-06 | 3,10E-03 |
| [P15586](https://www.uniprot.org/uniprot/P15586) | GNS | N-acetylglucosamine-6-sulfatase | -3,33E-05 | 1,09E-05 | 3,13E-03 |
| [Q9Y6X5](https://www.uniprot.org/uniprot/Q9Y6X5) | ENPP4 | Bis(5-adenosyl)-triphosphatase | -5,00E-05 | 1,64E-05 | 3,16E-03 |
| [O15240](https://www.uniprot.org/uniprotkb/O15240/entry) | VGF | Neurosecretory protein VGF | -3,73E-06 | 1,23E-06 | 3,20E-03 |
| [Q92752](https://www.uniprot.org/uniprot/Q92752) | TNR | Tenascin-R | -1,84E-05 | 6,10E-06 | 3,39E-03 |
| [Q14515](https://www.uniprot.org/uniprotkb/Q14515/entry) | SPRL1 | SPARC-like protein 1 | -3,40E-06 | 1,13E-06 | 3,55E-03 |
| [Q8WXD2](https://www.uniprot.org/uniprotkb/Q8WXD2/entry) | SCG3 | Secretogranin-3 | -3,44E-06 | 1,16E-06 | 4,03E-03 |
| [Q9UHC6](https://www.uniprot.org/uniprotkb/Q9UHC6/entry) | CNTP2 | Contactin-associated protein-like 2 | -1,28E-05 | 4,34E-06 | 4,12E-03 |
| [P17174](https://www.uniprot.org/uniprotkb/P17174/entry) | AATC | Aspartate aminotransferase, cytoplasmic | -1,22E-05 | 4,13E-06 | 4,15E-03 |
| [Q6MZW2](https://www.uniprot.org/uniprotkb/Q6MZW2/entry) | FSTL4 | Follistatin-related protein 4 | -1,22E-05 | 4,13E-06 | 4,20E-03 |
| [Q9HCU4](https://www.uniprot.org/uniprotkb/Q9HCU4/entry) | CELR2 | Cadherin EGF LAG seven-pass G-type receptor 2 | -3,60E-05 | 1,23E-05 | 4,34E-03 |
| [Q07954](https://www.uniprot.org/uniprotkb/Q07954/entry) | LRP1 | Prolow-density lipoprotein receptor-related protein 1 | -1,77E-05 | 6,11E-06 | 4,81E-03 |
| [Q9H2E6](https://www.uniprot.org/uniprotkb/Q9H2E6/entry) | SEMA6A | Semaphorin-6A | -2,03E-05 | 7,04E-06 | 4,91E-03 |
| [P04066](https://www.uniprot.org/uniprotkb/P04066/entry) | FUCO | Tissue alpha-L-fucosidase | -1,56E-05 | 5,43E-06 | 5,02E-03 |
| [P14415](https://www.uniprot.org/uniprotkb/P14415/entry) | AT1B2 | Sodium/potassium-transporting ATPase subunit beta-2 | -4,79E-05 | 1,66E-05 | 5,07E-03 |
