# Supplementary material for: Cerebrospinal fluid proteomic profile of frailty: Results from the PROLIPHYC cohort
Source: Aging Cell. 2024 May 2;23(7):e14168. doi: 10.1111/acel.14168 (PMC11258431; doi:10.1111/acel.14168)
Supplement: Supplementary file 3 — Data S3. [file ACEL-23-e14168-s002.docx]

**Supplemental Material**

**Frailty assessment**

A total of 40 variables were selected to measure the deficits in (1) executive functioning, (2) mood and cognition, (3) gait, (4) rigidity and agility of extremities, (5) blood laboratory tests of markers related to kidney function, metabolic function and inflammatory conditions, which are of particular importance in adults over 50 years old (Vásárhelyi & Debreczeni, 2017), (6) urinary symptoms, (7) nutrition and (8) history including medication intake and the presence of previously diagnosed hypertension or diabetes mellitus. Supplementary table 1 presents the variables and thresholds for deficit status. Following the guidelines (Searle, Mitnitski, Gahbauer, Gill, & Rockwood, 2008) for creating a FI, we did not include variables that had more than 5% missing data and less than 1% of participants meeting criteria for the deficit. Subjects presenting more than 10% of missing data on the 40 health deficits considered were excluded.

**Clinical characterization of the population**

The increased risk of AD was examined based on the ratio between CSF tau and amyloid beta 42 (Aβ42) peptides using a cut-off of 0.215 (Mo et al., 2017). Presynaptic dopamine deficiency, related to PD, was examined visually by an expert neurologist using [123I]FP-CIT DATSPECT. The presence of white matter lesions, which is a characteristic of increased risk of vascular dementia, was ascertained on FLAIR MRI images using the Fazekas scale with a cut-off of ≥ grade 2 (Fazekas, Chawluk, Alavi, Hurtig, & Zimmerman, 1987). Finally, abnormal CSF dynamics was quantified via the CSF outflow resistance R_0_ (Marmarou, Sawauchi, & Dunbar, 2005) derived from the lumbar infusion test (Vallet et al., 2020). A threshold of R_0_>12 mmHg/mL/min was chosen as a marker of altered CSF dynamics and increased risk of NPH. Indeed, the clinical symptoms mentioned above are not specific to NPH and may be associated with other neurodegenerative diseases. That is why the infusion test is recommended in clinical practice in order to characterize CSF dynamic alterations by a quantitative measurement of the resistance to CSF outflow R_0_.

Although the above in vivo markers do not, by themselves, corroborate the clinical diagnosis of neurodegenerative diseases, they do flag an increased risk of having or developing them. For each marker, the prevalence of subjects at risk was calculated only in subjects with available data.

**References**

Fazekas, F., Chawluk, J., Alavi, A., Hurtig, H., & Zimmerman, R. (1987). MR signal abnormalities at 1.5 T in Alzheimer’s dementia and normal aging. *American Journal of Roentgenology*, *149*(2), 351–356. https://doi.org/10.2214/ajr.149.2.351

Marmarou, A., Sawauchi, S., & Dunbar, J. (2005). Diagnosis and management of idiopathic normal-pressure hydrocephalus: A prospective study in 151 patients. *J. Neurosurg.*, *102*, 11.

Mo, Y., Stromswold, J., Wilson, K., Holder, D., Sur, C., Laterza, O., … Egan, M. F. (2017). A multinational study distinguishing Alzheimer’s and healthy patients using cerebrospinal fluid tau/Aβ42 cutoff with concordance to amyloid positron emission tomography imaging. *Alzheimer’s & Dementia: Diagnosis, Assessment & Disease Monitoring*, *6*(1), 201–209. https://doi.org/10.1016/j.dadm.2017.02.004

Searle, S. D., Mitnitski, A., Gahbauer, E. A., Gill, T. M., & Rockwood, K. (2008). A standard procedure for creating a frailty index. *BMC Geriatrics*, *8*(1), 24. https://doi.org/10.1186/1471-2318-8-24

Vallet, A., Del Campo, N., Hoogendijk, E. O., Lokossou, A., Balédent, O., Czosnyka, Z., … Schmidt, E. (2020). Biomechanical response of the CNS is associated with frailty in NPH-suspected patients. *Journal of Neurology*, *267*(5), 1389–1400. https://doi.org/10.1007/s00415-019-09689-z

Vásárhelyi, B., & Debreczeni, L. A. (2017). Lab Test Findings in the Elderly. *EJIFCC*, *28*(4), 328–332.
